# Early results of coronary artery bypass grafting with coronary endarterectomy for severe coronary artery disease

**DOI:** 10.1186/1749-8090-4-52

**Published:** 2009-09-22

**Authors:** Jan D Schmitto, Philipp Kolat, Philipp Ortmann, Aron F Popov, Kasim O Coskun, Martin Friedrich, Samuel Sossalla, Karl Toischer, Suyog A Mokashi, Theodor Tirilomis, Mersa M Baryalei, Friedrich A Schoendube

**Affiliations:** 1 Department of Thoracic-, Cardiac- and Vascular Surgery, Georg August University of Goettingen, Germany; 2 Department of Cardiology and Pneumology, Georg August University of Goettingen, Germany; 3 Department of Cardiac Surgery, Brigham and Women's Hospital, Harvard Medical School, Boston, MA, USA

## Abstract

**Background:**

Despite the existence of controversial debates on the efficiency of coronary endarterectomy (CE), it is still used as an adjunct to coronary artery bypass grafting (CABG). This is particularly true in patients with endstage coronary artery disease. Given the improvements in cardiac surgery and postoperative care, as well as the rising number of elderly patient with numerous co-morbidities, re-evaluating the pros and cons of this technique is needed.

**Methods:**

Patient demographic information, operative details and outcome data of 104 patients with diffuse calcified coronary artery disease were retrospectively analyzed with respect to functional capacity (NYHA), angina pectoris (CCS) and mortality. Actuarial survival was reported using a Kaplan-Meyer analysis.

**Results:**

Between August 2001 and March 2005, 104 patients underwent coronary artery bypass grafting (CABG) with adjunctive coronary endarterectomy (CE) in the Department of Thoracic-, Cardiac- and Vascular Surgery, University of Goettingen. Four patients were lost during follow-up. Data were gained from 88 male and 12 female patients; mean age was 65.5 ± 9 years. A total of 396 vessels were bypassed (4 ± 0.9 vessels per patient). In 98% left internal thoracic artery (LITA) was used as arterial bypass graft and a total of 114 vessels were endarterectomized. CE was performed on right coronary artery (RCA) (n = 55), on left anterior descending artery (LAD) (n = 52) and circumflex artery (RCX) (n = 7). Ninety-five patients suffered from 3-vessel-disease, 3 from 2-vessel- and 2 from 1-vessel-disease. Closed technique was used in 18%, open technique in 79% and in 3% a combination of both. The most frequent endarterectomized localization was right coronary artery (RCA = 55%). Despite the severity of endstage atherosclerosis, hospital mortality was only 5% (n = 5). During follow-up (24.5 ± 13.4 months), which is 96% complete (4 patients were lost caused by unknown address) 8 patients died (cardiac failure: 3; stroke: 1; cancer: 1; unknown reasons: 3). NYHA-classification significantly improved after CABG with CE from 2.2 ± 0.9 preoperative to 1.7 ± 0.9 postoperative. CCS also changed from 2.4 ± 1.0 to 1.5 ± 0.8

**Conclusion:**

Early results of coronary endarterectomy are acceptable with respect to mortality, NYHA & CCS. This technique offers a valuable surgical option for patients with endstage coronary artery disease in whom complete revascularization otherwise can not be obtained. Careful patient selection will be necessary to assure the long-term benefit of this procedure.

## Introduction

Fifty years ago, Bailey [1] was the first to describe coronary endarterectomy in man without cardiopulmonary bypass or associated coronary artery bypass grafting. Although no definitive conclusions could be drawn from his pioneering work, it was presented with the intent of encouraging further research in this unknown section. In the early years after this report, several institutions lead by Hallèn [2], Effler [3], Dilley [4] and many others shared his vision of long-term patency after revascularization and performed that procedure as an adjunct to CABG. The benefit was relief from angina in the majority of these cases; but the price to pay was higher incidence of postoperative mortality and morbidity. This fact has been the basis for several controversial debates [5,6]; proponents regarded this technique to be the last opportunity for patients with endstage atherosclerosis. Others have criticized the increased intra- and post-operative risks while questioning the long-term benefits [5]. Therefore, this add-on was only given status of second importance, limited for patients with high-grade atherosclerosis.

## Methods

One-hundred and four patients with diffuse coronary artery disease underwent coronary artery bypass grafting (CABG) with adjunctive coronary endarterectomy (CE) of at least one artery in the Department of Thoracic-, Cardiac- and Vascular Surgery, Goettingen. Data were analyzed with respect to mortality, functional capacity (NYHA) and angina pectoris (CCS). Information concerning the patients' preoperative status were extracted from an extensive clinical database. Postoperative data were obtained from our clinical database, as well as by telephone survey, postal questionnaire and physical examination. Actuarial survival was reported using Kaplan-Meyer analysis. Two different surgical techniques, the open and the closed coronary endarterectomy (CE) were used in this study. Both techniques involve making an incision in the coronary vessel to extract the atherosclerotic lesion. We defined the closed technique as creating an incision no longer than 2 cm proximal to the target for removing the plaque. Whereas, in the open technique an incision the length of the lesion is made to directly remove it. After surgery systemic heparinization was started early postoperatively to avoid thrombembolic complications or early occlusions of the coronaries. That means, if the postoperative bleeding rate was lower than 50 ml/hour we started with the heparine infusion already 4 hours postoperatively. 100 mg of aspirin were given daily, starting at the first postoperative day.

## Results

Details from this heterogeneous cohort are summarized in Table 1. Between August 2001 and March 2005, 104 patients underwent coronary artery bypass grafting (CABG) with adjunctive coronary endarterectomy (CE) in the Department of Thoracic-, Cardiac- and Vascular Surgery, University of Goettingen, Germany. Follow-up of 100 patients was complete: 88 patients were male, 12 were female, mean age was 65.5 +/- 9 years. Preoperative body mass index (BMI) was 28.33 ± 4.03 kg/m^2^, hypertension was present in 94%, 35 patients suffered from diabetes, 80% showed hypercholesterolemia, 54% were smokers and, finally, 55% had positive family history concerning the cardiovascular system.

**Table 1:** Preoperative patients characteristics

	**All**
**Patients (n)**	100
**Age (y)**	
Mean	65.5 ± 9.23
Range	35-83
**Cardiac Risk Factors**	
BMI (Mean)	28.33 ± 4.03
Hypertension	94
Diabetic mellitus	35
Hypercholesterolemia	80
(Ex-)Smoker	54
Family History	55
**Angina-Class**	
CCS I	20
CCS II	32
CCS III	31
CCS IV	15
Unknown	2
**Dyspnea**	
NYHA I	26
NYHA II	36
NYHA III	26
NYHA IV	10
Unknown	2
**Previous Stroke**	13
**Previous Myocardial Infarction**	50
**Previous PTCA**	14
**Operative Priority**	
Elective	2
Urgent	19
Emergency	50
Unknown	29
**Diseased vessels**	
x1	2
x2	3
x3	95

**Figure 1:**
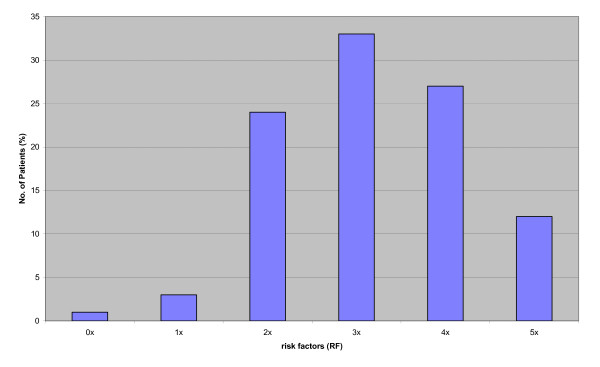
**Distribution of risk factors**.

Each patient could be associated with more than 3 cardiac risk factors (mean: 3.2 ± 1.1); patients with 2 or less risk factors represented the minority, as shown in Figure 1.

Ninety-five patients suffered from 3-vessel-disease, 3 patients from 2-vessel- and 2 patients from 1-vessel-disease. Referring to the Canadian Cardiovascular Society (CCS), 20% were preoperatively considered to belong to CCS I, 32% in II, 31% in III and 15% in IV. The cohort was also distributed referring to New York Heart Association criteria (NYHA): 26% were NYHA I, 36% NYHA II, 26% belong to NYHA III and 10% to NYHA IV.

To underline severity of atherosclerosis, previous events like stroke (13%), myocardial infarction (50%) and prior PTCA (14%) were also considered. Forty percent of the cases were given an operative priority of an emergency procedure. Detailed information concerning the distribution of gender among cardiac risk factors is shown in Table 1. Three-hundred and ninety-six vessels were bypassed (4 ± 0.9 vessels per patient). In 98% of cases, the left internal thoracic artery (LITA) was used as arterial bypass graft and a total of 114 vessels were endarterectomized. CE was performed on right coronary artery (RCA) (n = 55), on left anterior descending artery (LAD) (n = 52) and circumflex artery (RCX) (n = 7).

Closed technique was used in 18%, open technique in 79% and in 3% a combination of both. Cardiopulmonary bypass time was 192 ± 56 minutes, aortic cross clamp time was 119 ± 32 minutes. Three myocardial infarctions occured during operation time, defined by ST-elevations in electrocardiography and postoperative CK-(> 270 U/l)/CKMB-(>17 U/l) levels (Table 2). Duration of intensive care unit (ICU) stay was 5.6 ± 8.4 days. Ventilation was obtained for 52.9 ± 100.8 hours for the whole cohort. Isolating the smokers group, artificial respiration time was 61.7 ± 125.5 hours; non-smokers had to be supported for 41.7 ± 54.1 hours, more than 48% less.

**Table 2:** Operative data

**Number of Grafts**	
x1	1
x2	5
x3	19
x4	46
x5	24
x6	4
Unknown	1
Mean	4 ± 0.95
	
**Number of CE**	
RCA	55
LAD	35
Cx	7
DB	17
	
**Cross Clamp Time (min)**	119 ± 31.6
**Cardiopulm. Bypass Time (min)**	192 ± 56.5

Infection rate (as defined by leucocytosis, temperature >38°C and C-reactive protein >8 mg/l) on ICU was 18%, main complications were bronchopulmonary (n = 18), sternal infection (n = 3) and sepsis (n = 2). Multiple matches per patient were possible. Despite severity of endstage atherosclerosis hospital mortality was only 5% (n = 5). During follow-up (24.5 ± 13.4 months), which is 96% complete (4 patients were lost caused by unknown address), 8 patients died (cardiac failure: 3; stroke: 1; cancer: 1; unknown reasons: 3). All deaths, except one, were male patients. Mean follow-up-time, this study is based on, was 24.5 ± 13.4 months after surgery.

**Figure 2:**
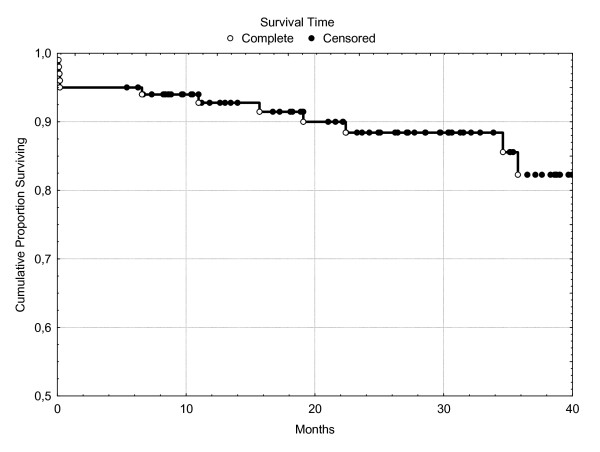
**Kaplan-Meyer-Survival-Curve**.

Patients were discharged from hospital after 15.9 ± 13.9 days. Survival rates for all patients are presented in Kaplan-Meyer curve in Figure 2. NYHA-classification clearly improved after CABG with CE from 2.2 ± 0.9 preoperative to 1.7 ± 0.9 postoperative. 48 patients were postoperatively regarded to belong to NYHA I, 30 to NYHA II, 4 to NYHA III and finally, 8 to NYHA IV. Preoperative distribution was 26% for NYHA I, 36% NYHA II, 26% belong to NYHA III and 10% to NYHA IV. CCS also changed from 2.4 ± 1.0 to 1.5 ± 0.8. 58 patients were regarded to belong to CCS I, 21 to CCS II, 6 to CCS III and 3 to CCS IV. Preoperatively, 20% were considered to belong to CCS I, 32% in II, 31% in III and 15% in IV.

## Discussion

Coronary artery bypass grafting is a worldwide routine cardiac surgery procedure to revascularize ischemic myocardium of patients with severe coronary artery disease. The operation was performed over 51,000 times in Germany in the year 2006 [7]. Due to demographic development, an increasing number of elderly and patients with multiple co-morbidities, improvements in cardiology diagnostics, medications and invasive interventions (PTCA or stenting), cardiac surgeons often are confronted with geriatric patients suffering from diffusely and severely calcified coronary artery disease. While the total number of operative interventions has decreased, the complexity and severity of each procedure has increased. In addition, elderly patients with diffusely calcified multi-vessel atherosclerosis, especially of the smaller branches, are not amenable to stenting and angioplasty - cardiologic methods are limited. Therefore, it is important to offer a valuable alternative to these patient that begins at the point where the possibilities of conservative medicine end.

Since Bailey's first coronary endarterectomy in the late 50's [1], the circumstances under which cardiac operations are performed have since changed, including: the use of cardiopulmonary bypass, increased technical and pharmaceutical improvements and last but not least, the growing experience of cardiac surgeons led to a point, in which procedures like these can be performed more safely and can almost be considered to be routine, just like CABG itself. Today's conditions are not comparable with those of starter-time, in which controversial debates on efficiency of coronary endarterectomy (CE) were held. Higher rates of morbidity and mortality were the frequent points of criticism and forced CE to play a role of second importance [5,6]. Therefore, it is important to focus on current results, to rethink of this alternative and to reevaluate the indication for this surgical technique [5,6].

In our department, coronary endarterectomy combined with CABG was performed in 104 cases in about four years. Indication for CE was handled restrictively. It was only performed on occluded, nearly occluded and/or calcified vessels with long-range stenoses, if regular anastomosis between graft and coronary artery seemed to be technically impossible. Decision to perform endarterectomies was therefore made intraoperatively, based on local findings on these patients with severe calcified atherosclerosis. The operations were performed by fully-trained staff cardiac surgeons. Although a surgical trainee participated in the operations, the critical portions were performed by experienced surgeons.

This severity of atherosclerosis that affects the coronary arteries and its fatal consequences may be demonstrated through following findings: in the majority of the cases, an event like stroke or myocardial infarction occurred before intervention. According to NYHA and CCS it is understandable that these patients were hardly able to handle daily life activities. This was not only revealed by the NYHA-/CCS-constellation or preoperative symptoms like vertigo, edema or syncopes but also by interviews and postal questionnaires. In 40% of the cases, CABG and CE were performed as an emergency procedure -one more clue for the progressed severity of this disease.

Our results also indicate an expected match among this heterogeneous group of patients: nearly all of them, except one woman, show a variegated mixture of cardiac risk factors (Figure 1), averaging more than three factors per patient.

Even today, in the era of wide spread information concerning health-prevention via different media, these disease-supporting avoidable factors are still present and imply a major difficulty in coping with the disease.

Operation time and therefore cardiopulmonary bypass- as well as aortic cross clamp time were longer comparing with other studies. This can be explained by several factors:

1. Operation technique: in most cases open technique was used based on surgeons' preferences. It offers free insight to local findings, exposing the whole arterial lumen and side branches containing atherosclerotic occlusive material and therefore guarantees the quality (which means avoiding an intima flap and therefore assure the same plane etc.) of the endarterectomy by avoiding residuals. On the other hand it takes more time to fulfil the long-range suture of the vessel.

2. Severity of atherosclerosis: many vessels are deeply affected in this cohort. The majority suffered from three-vessel-disease, resulting in almost four bypass-grafts per patient.

3. Localization: a huge amount of vessels that were bypassed or/and endarterectomized belonged to right coronary artery system, which is probably technically the most challenging localization. Preparation as well as suturing on the back side of heart is time-consuming.

4. High rate of calcification: CE was only performed, when regular anastomosis seemed to be impossible. It was meant to be the last possibility to revascularize ischemic myocard. The high number of CE's performed underlines the fact of endstage coronary artery disease among our patients.

5. LITA was used in 98%: LITA is still the most important graft that can be used in order to guarantee long-term-patency caused by better vasomotor function concerning the flow-rate [5-8]. In case of bypassing more than one vessel, which was the common case, veins like vena saphena magna or parva were used, as usually.

The duration of time spent in the ICU is greater in our study as compared to other studies [8-10], but this is based on the fact that an intermediate-care unit was interposed in our hospital. Patients from the ICU were directly sent directly to the ward floor, once their general health status was deemed appropriate. According to the fact that our patients suffered from severe endstage atherosclerosis as well as from other typical geriatric morbidities, it is not surprising that the length of stay is prolonged.

Hospital-mortality-rate, as presented in our study, is acceptable compared to other studies which range from 2.0 - 6.5% [5,6,8,11-16], considering the preoperative health state of the patients. It is remarkable that every death, with the exception of one, involved males. Following revascularization by CABG and adjunctive CE, the patients' subjective state of health significantly improved.

No reports exist addressing the issue of ventilation time on CE in the ICU. In our study, we have interestingly shown that the span between smokers and non-smokers is remarkable. Non-smokers required 48% less time with ventilator assistance compared to the former with a positive nicotine history. This corresponds to a lower risk for tracheal irritation and broncho-pulmonary infection. Similar results for CE patients are not described in literature so far. Further research on this topic seems to be necessary.

Despite the controversy surrounding the efficiency of coronary endarterectomy (CE) [5,6,17-20], it is still used as an adjunctive treatment to coronary artery bypass grafting (CABG) for patients with highly calcified, end-staged coronary artery disease.

## Conclusion

This study demonstrates that results of coronary endarterectomy are acceptable with respect to mortality, NYHA and CCS. CABG and adjunctive CE offers a valuable surgical option for patients with endstage coronary artery disease in whom complete revascularization otherwise could not be obtained. It is important to note, however, that this technique should not be considered a substitute for CABG. Not every patient undergoing bypass is suitable for this procedure. The procedure should be performed by highly experienced surgeons. Nevertheless, the reporting of additional experience and follow-up data will be necessary to assure long-term benefits.

## Competing interests

The authors declare that they have no competing interests.

## Authors' contributions

JDS conceived of the study, and participated in its design and coordination. PK conceived of the study, and participated in its design and coordination. PO participated in the design of the study and performed the statistical analysis. AFP conceived of the study, and participated in its design and coordination. KOC participated in the design of the study and performed the statistical analysis. MF participated in the design of the study and performed the statistical analysis. SS conceived of the study, and participated in its design and coordination. SAM participated in the design of the study and performed the statistical analysis. TT participated in the design of the study and performed the statistical analysis. MMB participated in the design of the study and performed the statistical analysis. FAS conceived of the study, and participated in its design and coordination. All authors read and approved the final manuscript.
